# Does Antenatal Maternal Psychological Distress Affect Placental Circulation in the Third Trimester?

**DOI:** 10.1371/journal.pone.0057071

**Published:** 2013-02-20

**Authors:** Anne Helbig, Anne Kaasen, Ulrik Fredrik Malt, Guttorm Haugen

**Affiliations:** 1 Norwegian Resource Centre for Women’s Health, Oslo University Hospital, Oslo, Norway; 2 Department of Obstetrics, Oslo University Hospital, Oslo, Norway; 3 Department of Health, Nutrition and Management, Oslo and Akershus University College of Applied Sciences, Oslo, Norway; 4 Department of Neuropsychiatry and Psychosomatic Medicine, Oslo University Hospital, Oslo, Norway; 5 Institute of Clinical Medicine, University of Oslo, Norway; VU University Medical Center, Netherlands

## Abstract

**Introduction:**

Some types of antenatal maternal psychological distress may be associated with reduced fetal growth and birthweight. A stress-mediated reduction in placental blood flow has been suggested as a mechanism. Previous studies have examined this using ultrasound-derived arterial resistance measures in the uterine (UtA) and umbilical (UA) arteries, with mixed conclusions. However, a reduction in placental volume blood flow may occur before changes in arterial resistance measures are seen. Fetoplacental volume blood flow can be quantified non-invasively in the umbilical vein (UV). Our objective was to study whether specific types of maternal psychological distress affect the placental circulation, using volume blood flow quantification in addition to arterial resistance measures.

**Methods:**

This was a prospective observational study of 104 non-smoking pregnant women (gestational age 30 weeks) with uncomplicated obstetric histories. Psychological distress was measured by General Health Questionnaire-28 (subscales anxiety and depression) and Impact of Event Scale-22 (subscales intrusion, avoidance and arousal). UtA and UA resistance measures and UV volume blood flow normalized for fetal abdominal circumference, were obtained by Doppler ultrasound.

**Results:**

IES intrusion scores above the mean were associated with a reduction in normalized UV volume blood flow (corresponding to –0.61 SD; *P* = 0.003). Adjusting for UA resistance increased the strength of this association (difference –0.66 SD; *P*<0.001). Other distress types were not associated with UV volume blood flow. Maternal distress was not associated with arterial resistance measures, despite adjustment for confounders.

**Conclusions:**

Intrusive thoughts and emotional distress regarding the fetus were associated with reduced fetoplacental volume blood flow in third trimester. Uterine and umbilical artery resistance measures were not associated with maternal distress. Our findings support a decrease in fetoplacental blood flow as a possible pathway between maternal distress and reduced fetal growth.

## Introduction

The impact of maternal psychological distress during pregnancy on offspring physical and mental health outcomes has been the subject of extensive research during the past decades. There is increasing evidence from animal and human studies indicating that antenatal stress, anxiety, and depression adversely affect various pregnancy and infant developmental outcomes [1]–[5]. Psychological distress has been suspected of causing reduced fetal growth and birthweight, although human studies have not shown consistent results [4], [6], [7]. Partially, this may due to the considerable methodological challenges and variations involved [5], [6], [8]. Recent reviews conclude that a negative effect of some types of psychological distress on birthweight seems to be present, but small [6], [7]. Two well-controlled prospective population-based studies report birthweight reductions of 20–50 g [9], [10]. While the effect size may not be of clinical relevance, it represents a physiological event and may be part of the complex interplay between the maternal environment, the placenta, and the developing fetus.

The biological mechanisms behind the possible association of maternal distress and fetal growth are unclear. A reduction in placental blood flow due to vasoactive properties of maternal stress hormones has been proposed as a possibility [1], [3], [11]. This could lead to a reduction in nutrients and oxygen delivered from the placenta to the fetus. Placental blood flow can be assessed non-invasively, on the maternal side in the uterine arteries (UtA), and on the fetal side in the umbilical vessels (see Figure S1, Schematic drawing of the placental circulation). Doppler ultrasound renders a graphic representation of blood flow velocity waveforms in the insonated vessel. Semi-quantitative resistance indices such as the pulsatility index (PI) are commonly calculated. These describe ratios between blood flow velocities in different parts of the heart cycle. Increased vascular resistance in the placental microcirculation leads to lower velocities, particularly during diastole, resulting in a higher PI. Diastolic notching in the UtA velocity waveform may also be observed in increased vascular resistance. Increased resistance indices in the UtA or UA as well as UtA notches are known to be associated with fetal growth restriction and the development of preeclampsia [12], [13].

Previous studies have investigated the association between psychological distress and the arterial resistance indices on the maternal and fetal side of the placental circulation in third trimester, yielding mixed results [11], [14]–[16]. However, while these resistance indices are easy to obtain, they are semi-quantitative and provide only indirect estimates of placental vascular resistance. Their ability to detect moderate changes in placental volume blood flow is limited [17]. Volume flow quantification provides more direct physiological information [18], [19]. Since the umbilical vein (UV) is the single vessel leading oxygen and nutrient-rich blood from the placenta to the fetus, it is uniquely suited to measure fetoplacental volume blood flow. UV volume blood flow (Q_UV_) is closely associated with fetal growth [17], [19]–[21]. Q_UV_ normalized for fetal size has been shown to decrease up to several weeks before fetal biometry [20] or UA resistance indices indicate growth restriction and placental compromise [17] [22]. The correlation between UA resistance indices and Q_UV_ is only mild to moderate [23], [24]. Therefore, the more sensitive Q_UV_ could be a useful measure when investigating the role of fetoplacental blood flow in the possible association between maternal distress and reduced fetal growth. To our knowledge, this has not been studied before.

Extensive research indicates that different types of antenatal distress or stressors, e.g. general anxiety, pregnancy-specific anxiety, depression, chronic strain, and life events, have differential effects on pregnancy and infant developmental outcome [5], [25]. Therefore, examining the effects of different types of psychological distress on the placental circulation is of relevance.

We aimed to examine whether several key types of maternal psychological distress in the third trimester affect the placental circulation, by using arterial resistance measures on the maternal and the fetal side of the placenta, as well as fetoplacental volume blood flow quantification. We expected that antenatal distress would be more strongly correlated with volume blood flow than with the arterial resistance indices.

## Materials and Methods

### Ethics Statement

The study was approved by the Regional Committee for Medical Research Ethics, Southern Norway, Oslo, Norway (S-05281). Written informed consent was obtained from all participants, and the study was conducted according to the Declaration of Helsinki.

### Methods

The present study is part of a larger prospective study investigating psychological distress in three groups of pregnant women, with either a current diagnosis of fetal malformation, malformation in previous offspring, or no history of malformation. The present study describes only the group with no previous or current fetal malformations. Design, choice of psychometric tools, and sample size of the different groups was determined with regard to the larger study [26].

### Participants

Between April 2007 and February 2009, 111 pregnant women booked for delivery at our hospital were included consecutively, but with limitations imposed by workload (i.e. convenience sampling). They were invited after a routine second trimester ultrasound scan with normal results. Exclusion criteria were multiple pregnancy, insufficient fluency in Norwegian, history of severe obstetric complications, history of a previous fetus or child with a structural or developmental disorder, overt psychiatric disorders (e.g. severe bipolar disorder, psychosis, drug abuse), and age below 18 years.

At 30 weeks gestation, participants met for an assessment including psychometric questionnaires and ultrasound examination. Weight was measured, and body mass index (BMI; kg/m^2^) was calculated using self-reported height. Sociodemographic, medical, and obstetric data were systematically collected by interviews, self-report questionnaires and from medical records. After birth neonates were weighed within two hours, using the same calibrated electronic scale.

#### Psychometric measures

Psychological distress was assessed by two self-report questionnaires: the 28*-*item version of the General Health Questionnaire (GHQ*-*28) [27] and the 22*-*item Impact of Event Scale (IES*-*22) [28], [29].The GHQ-28 has been widely used to estimate the prevalence of mental disorder in a given population, but also as a measure of psychological distress and subjective well-being in clinical and non-clinical populations. It has also been used in relation to pregnancy and childbirth [26], [30], [31]. All items have four possible responses and emphasize the last two weeks. There are different methods to score GHQ-28, the Likert method and the so-called case score method. Likert scoring (item scores 0–1–2–3, scoring range 0–84 for the sum score) is mostly used as an ordinal scale to measure level of distress. If this method is used to estimate prevalence of probable clinically significant levels of distress, the mean score in a given population is suggested to be indicative of the best threshold [32]. Other authors suggest that the median score or stratum-specific likelihood ratios are better parameters [33]. The binary case score method uses 0–0–1–1 scoring per item (total range 0–28). If used to estimate the prevalence of clinically significant distress, the binary method is mostly used with a cut-off level ≥6.

Factor analyses of the GHQ-28 have identified four subscales, with seven items each. The anxiety subscale addresses symptoms such as sleep problems, nervousness and panic attacks. The depression subscale deals with rather severe symptoms such as hopelessness and suicidal ideation. The somatic symptoms subscale covers general health issues, e.g. headaches, tiredness and health perception. Social dysfunction focuses on general well-being and quality of life, e.g. satisfaction with the way one carries out ones tasks and ability to enjoy normal day-to-day activities. We considered the anxiety and depression subscales to be most relevant for our specific research question. Likert scoring was used, providing a scoring range of 0–21 for each subscale. GHQ-28 subscales are inter-correlated and mostly used as ordinal scales to measure severity of symptom dimensions, not as case-finders for e.g. clinical depression or anxiety.

GHQ-28 addresses psychological distress and psychiatric symptoms in general. In order to cover pregnancy-specific distress as well, we applied the Impact of Event Scale (IES). IES measures symptoms of distress occurring during the previous week, in relation to specific stressful or traumatic events. It has good psychometric properties in both clinical and non-clinical samples [34] and has been used in several studies on pregnancy and postpartum distress [26], [30], [31], [35]. In the present study the questionnaire referred to “the condition of the child” as a possible cause for distress. The IES*-*22 consists of 22 items (scoring range 0–5 per item) and has three subscales. Intrusion (seven items, range 0–35) deals with symptoms such as intrusive and unbidden thoughts, emotions, dreams and memories. Avoidance (seven items, range 0–35) addresses emotional numbness, denial, and avoiding stimuli or thoughts related to the health of the fetus. Arousal (eight items, range 0–40) focuses on psycho-physiological symptoms such as hypervigilance, irritability and heightened startle response. In clinical samples, subscale scores below 9 usually indicate minor responses, 9–19 moderate responses, and above 19 clinically important responses.

Physiological changes may also occur within the “normal” range. Therefore, in this study we chose to use continuous scores as well as a cut-off level at the mean as suggested by Goldberg *et al*. [32], for all psychometric scores.

#### Ultrasonography

The ultrasound examination was done immediately after completion of the questionnaires, by an experienced operator (AH or GH), using an Acuson Sequoia 512 ultrasound machine (Mountain View, CA, USA) with a transabdominal 2.5–6 MHz curvilinear probe. Abdominal circumference, UV diameter, and blood flow velocity waveforms of both UtAs, one UA, and the intra-abdominal UV were obtained with participants in a semi-recumbent position (see Figure S1, Schematic drawing of the placental circulation). Flow velocity waveforms were acquired using pulsed-wave Doppler with color Doppler guidance. High pass filter was set at 50 Hz. The sample volume was adjusted to encompass the diameter of the vessel, and the lowest possible insonation angle was used. The insonation angle was always below 25 degrees, and angle correction was applied. Images were stored digitally and analyzed off-line by the ultrasonographer, blinded to distress scores. Acoustic output and exposure time was kept as low as reasonably achievable.For the UtA, the sample gate was placed within 1 cm ventrally of the cross-over with the external iliac artery. The UA was assessed in a free loop of the umbilical cord during fetal quiescence. Three consecutive blood flow velocity waveforms in steady state were manually traced and their mean PI calculated. For the UtA, the average of both sides was used. If only one UtA was identified, the unilateral mean PI was used (*n = *3). Maternal and fetal heart rates were obtained as an average of three waveforms from the right UtA and the UA, respectively. A UtA diastolic notch was defined as a definite increase in velocity after the initial fall at the end of systole. If this was minor, the image was assessed independently by both ultrasonographers. In the four cases of discrepancy, notches were scored as absent.

UV flow velocity waveforms were obtained during fetal quiescence from the intra-abdominal straight part of the vessel. Steady state waveforms of 2–4 seconds duration were traced manually, and the time-averaged maximum velocity (V_TAMX_, cm/s) recorded. UV inner diameter was measured at the same site, perpendicular to the vessel wall, in memory buffer frames with the best visualization of the vessel walls. The mean was calculated from at least five measurements [36]. Fetal abdominal circumference (AC, cm) was measured in a standardized transverse view and recorded as the mean of three measurements. Q_UV_ (ml/min) was calculated as (0.5 * V_TAMX_ * π* (UV diameter/2)^2^ * 60) [37] and divided by fetal AC to normalize for fetal size (Q_UVAC_, ml/min/cm). Normalizing for AC instead of estimated fetal weight was chosen in order to minimize measurement errors by using a single rather than a composite measure [17], [19], [37].

#### Statistical analysis

Main outcome variables were UtA PI, UA PI, and Q_UVAC_. Secondary outcome variables were UtA notching (both bilateral and “any” notch), maternal and fetal heart rates. Explanatory variables were the different distress measure scores, continuous as well as dichotomized at the mean. For regression models, covariates gestational age, maternal age, parity (para 0/≥1), assisted fertilization (yes/no), BMI, fetal gender, fetal and maternal heart rates, and ultrasonographer were selected *a priori*, based on previous research. For descriptive statistics and comparisons between groups parametric or non-parametric analyses were used as appropriate. UtA PI and Q_UVAC_ were not normally distributed, and ln-transformed if this allowed the use of parametric methods. Bivariate associations between variables were explored by cross-tables and scatter plots with Pearson’s or Spearman’s correlation coefficients, as appropriate. Linear regression analyses were performed for the primary outcome measures. Distress measures and other covariates were first examined in univariate linear regression analyses. Those with *P*<0.20 were entered stepwise into multiple regression models, and variables with an adjusted *P*<0.10 were kept in the final models. Distress measures were analyzed separately. Model assumptions regarding normality, linearity, outliers, homoscedasticity and independence of residuals were checked. Sample size was determined by the larger study which this subset of women participated in. The current sample was sufficient to detect a difference of 0.65 SD between two similarly sized groups with a power of 90% and a 5% significance level.

Data analysis was performed using SPSS version 18 (Statistical Package for the Social Sciences, Chicago, IL, USA). A *P*<0.05 was considered statistically significant.

## Results

Of the 111 women originally recruited, 107 met at 30 weeks gestational age (two withdrawals, two had scheduling problems). Three participants were excluded for the following reasons: hypertensive disease before 20 weeks (*n = *1), medication linked to reduced fetal growth (*n = *1), and smoking (*n = *1). There were no cases of gestational diabetes or other chronic diseases known to affect fetal growth.

In the remaining 104 pregnancies, Doppler measurements were obtained in all for UtA (101 bilateral, three unilateral) and UA, and in 96 for Q_UVAC_. Reasons for not succeeding were fetal activity or position.

Population characteristics and obstetric outcome are described in Table 1. Of the five women who developed pregnancy-induced hypertension (PIH) or late-onset preeclampsia during pregnancy (PE), only one had hypertension at the 30 week assessment.

**Table 1:** Maternal and infant characteristics (*n = *104).

Characteristic	Value
Maternal age (years)	31.5±4.1
Parity ≥1	45 (43)
Body mass index (kg/m^2^) at 30 weeks	26.2 (24.6–28.4)
Assisted fertilization (yes)	7 (7)
Gestational age at assessment (weeks)	30.0±0.6
Pregnancy induced hypertension/preeclampsia	5 (5)*
Gestational age at delivery (weeks)	40.3 (39.4–41.0)
Preterm birth (<37 weeks)	4 (4)
Birthweight (grams)	3551 (3326–3842)
Fetal gender (female)	62 (60)

Data are given as mean ± SD, median (interquartile range) or *n* (%).

* One pregnancy-induced hypertension at 30 week assessment, all others later onset.

Distress measures are presented in Table 2. Most women had relatively low GHQ-28 sum scores, with very low scores on depressive symptoms, but higher on the anxiety subscale. IES subscale scores were relatively low, with the exception of intrusion subscale scores, where 36% scored above the cut-off level for a moderate response.

**Table 2:** Psychological distress scores at 30 weeks of gestational age (*n* = 104).

	Mean (± SD)	Median (Range)	Cut-off level*	Prevalence above cut-off level, n (%)
**General Health Questionnaire**				
Sum Likert score	18.5±7.4	17 (7–49)	score ≥ mean	47 (45)
Sum case score	3.8±4.1	2.0 (0–21)	score ≥6	27 (26)
Anxiety	4.7±2.8	4 (0–15)		
Depression	0.2±0.7	0 (0–5)		
Somatic symptoms	5.6±2.4	5 (0–16)		
Social dysfunction	8.0±2.4	7 (0–18)		
**Impact of Event Scale**				
Intrusion	6.8±6.6	5 (0–31)	score ≥9	37 (36)
			score ≥20	3 (3)
Avoidance	1.4±3.3	0 (0–22)	score ≥9	4 (4)
			score ≥20	1 (1)
Arousal	3.6±3.8	2 (0–21)	score ≥9	13 (12)
			score ≥20	1 (1)

Psychometric scores and prevalence of different levels of psychological distress in the study population.

* For choice of cut-off levels for psychological distress, see methods.

Circulatory outcome measures are described in Table 3. There were no cases of absent or reversed enddiastolic flow in the UA.

**Table 3:** Circulatory outcome measures.

	n	Value
**Maternal**		
Uterine artery PI	104	0.72 (0.61–0.80)
Any notch	104	25 (24)
Bilateral notch	101	7 (7)
Maternal heart rate (bpm)	104	83.6±10.9
**Fetal**		
Umbilical artery PI	104	1.03±0.17
Fetal heart rate (bpm)	104	141.6±9.0
Q_UV_ (ml/min)	96	133.9 (107.1–160.1)
Q_UVAC_ (ml/min/cm)	96	4.93 (4.21–6.03)

Data are given as median (interquartile range), *n* (%), or mean ± SD.

bpm, beats per minute; Q_UV_, umbilical vein volume blood flow; Q_UVAC_, umbilical vein volume blood flow normalized for fetal abdominal circumference; PI, pulsatility index.

### 

#### Uterine arteries

We found no significant correlations between any of the psychometric scores and UtA PI or maternal heart rate (results not shown). Scatter plots did not indicate any non-linear pattern of correlation. The prevalence of notches (bilateral notch, or presence of any notch) was not significantly different in those below or above mean distress scores.

Linear regression analyses were performed with UtA PI as outcome variable, examining distress measures and other covariates as described above. The only distress measures or covariates reaching *P*<0.20 were intrusion dichotomized by mean (*P* = 0.176), and maternal heart rate (*P = *0.057). The multiple linear regression model using these two variables was not statistically significant.

#### Umbilical artery

None of the psychometric scores were significantly correlated with UA PI or fetal heart rate (results not shown). Scatter plots did not show any non-linear patterns of correlation. No association with dichotomized distress scores was found.

In univariate linear regression analyses with UA PI as outcome variable, variables reaching *P*<0.20 were intrusion dichotomized by mean (*P = *0.113), fetal heart rate (*P*<0.001), gestational age (*P = *0.094) and ultrasonographer (*P = *0.077). In a multiple regression model with these variables, only gestational age and FHR reached *P*<0.10.

### Umbilical Vein Volume Blood Flow

Q_UVAC_ was significantly correlated with intrusion (*r*_s_ = –0.22, *P = *0.031) (see Figure S2, Scatter plot showing correlation between IES intrusion scores and fetoplacental volume blood flow (Q_UVAC_)), but not with any other distress measure. Intrusion dichotomized at the mean (i.e. 6.8) showed a significantly lower Q_UVAC_ in the more distressed group (mean difference = –0.791 ml/cm/min; 95% CI –1.319, –0.264; *P = *0.004).

In univariate regression analyses, the only distress score meeting inclusion criteria was intrusion. Covariates with *P*<0.20 were maternal age and fetal gender (see Table 4). In multiple regression models, keeping only covariates with an adjusted *P*<0.10 (i.e. maternal age), intrusion as a continuous variable was statistically significant (*P = *0.032) and uniquely explained 4% of Q_UVAC_ variance. For intrusion dichotomized at the mean, the *P*-value was 0.003 and explained 9% variance (see Table 5). The effect estimate for the dichotomous variable corresponded to 0.61 SD. The regression models explained 5% and 9% of the total Q_UVAC_ variance and were statistically significant (*P*-values 0.038 and 0.004, respectively).

**Table 4:** Associations between non-psychometric covariates and normalized umbilical vein volume blood flow (*n* = 96).

	unadjusted B	95% CI	P
Maternal age (years)	0.046	–0.019, 0.112	0.165
Parity (≥1)	0.189	–0.362, 0.739	0.50
BMI at 30 weeks gestation	–0.043	–0.131, 0.045	0.34
Assisted fertilization (yes)	0.483	–0.566, 1.531	0.36
Gestational age (weeks)	–0.171	–0.273, 0.620	0.45
Fetal gender (girl)	–0.374	–0.926, 0.178	0.181
Ultrasonographer	0.347	–0.509, 1.203	0.42
Fetal heart rate (bpm)	0.017	–0.013, 0.048	0.26
Umbilical artery PI	–2.444	–4.033, –0.856	0.003
Uterine artery mean PI	0.807	–0.504, 2.119	0.22

Results from univariate linear regression with normalized umbilical vein volume blood flow (Q_UVAC_; ml/min/cm) as outcome variable.

B, regression coefficient; BMI, body mass index; bpm, beats per minute; PI, pulsatility index.

**Table 5:** Determinants of normalized umbilical vein volume blood flow, with continuous or dichotomized distress score (*n = *96).

	unadjusted			adjusted for maternal age				adjusted for maternal age and UA PI			
	B	95% CI	P	B	95% CI	β	P	B	95% CI	β	P
**A. Continuous distress measure**											
IES intrusion	–0.039	–0.079, 0.001	0.053	–0.044	–0.084, –0.004	–0.220	0.032	–0.045	–0.083, –0.007	–0.226	0.020
Maternal age (years)	0.046	–0.019, 0.112	0.165	0.056	–0.009, 0.121	0.171	0.093	0.064	0.002, 0.126	0.196	0.044
Umbilical artery PI	–2.444	–4.033, –0.856	0.003					–2.583	–4.123, –1.042	–0.318	0.001
**B. Dichotomous distress measure**											
IES intrusion>mean	–0.791	–1.319, –0.264	0.004	–0.814	–1.338, –0.291	–0.302	0.003	–0.879	–1.370, –0.382	–0.325	0.001
Maternal age (years)	0.046	–0.019, 0.112	0.165	0.052	–0.012, 0.115	0.159	0.108*	0.060	0.001, 0.120	0.186	0.047
Umbilical artery PI	–2.444	–4.033, –0.856	0.003					–2.728	–4.220, –1.237	–0.335	<0.001

Results from univariate and multiple linear regression showing associations between distress measure IES intrusion and umbilical vein volume blood flow normalized for fetal abdominal size (Q_UVAC_; ml/min/cm). IES intrusion scores are continuous in section A, and dichotomized at the mean in section B.

* Maternal age does not fulfill criteria of P<0.10 for continued inclusion into this regression model, but is retained for easier comparison with section A.

B, regression coefficient; β, standardized regression coefficient; IES, Impact of Event Scale; UA, umbilical artery; PI, pulsatility index.

UA PI was significantly correlated with Q_UVAC_ (*r* = –0.30, *P* = 0.003). To explore a possible mediating effect of placental vascular resistance, we included both UA PI and intrusion as predictors in multiple regression models with Q_UVAC_ as dependent variable. Other covariates were tested and included as previously described. The strength of the association between intrusion scores and Q_UVAC_ increased (see Table 5). Continuous intrusion scores explained 5% of Q_UVAC_ variance (*P* = 0.20). For intrusion above the mean, the *P*-value was 0.001 and explained variance 10% (see Table 5). The effect estimate corresponded to 0.66 SD for intrusion scores above the mean. The resulting models were highly significant (*P*-values 0.001 and <0.001, respectively) and explained 14 and 20% of Q_UVAC_ variance. Interestingly, the standardized regression coefficients for intrusion and UA PI were of similar magnitude (see Table 5). The association between intrusion and Q_UVAC_ decreased slightly after removal of the five participants who subsequently developed PIH/PE, but remained significant (adj. *B* = –0.843; 95% CI –1.364, –0.322; *P* = 0.002 for scores above the mean).

## Discussion

We aimed to investigate the effect of several key types of maternal psychological distress on the maternal and the fetal side of the placental circulation in third trimester, using arterial blood flow resistance measures in the UtA and the UA, as well as fetoplacental volume flow quantification in the UV. While we did not find any association with UtA and UA PI, the psychometric score indicating intrusive emotional distress concerning the health of the fetus was negatively correlated with normalized fetoplacental volume blood flow, i.e. Q_UVAC_. This supports our hypothesis that maternal psychological distress is more strongly correlated with fetoplacental volume flow than with arterial resistance measures.

Some studies have reported increased UtA resistance measures related to maternal distress in third trimester [11], [16]. However, our negative findings are in agreement with the larger study by Harville *et al.* [15], which did not confirm this association. Regarding UA resistance measures in third trimester, our negative results concur with most earlier studies [11], [15], [16]. Only one study consisting of 37 patients has reported an increased UA PI in anxious women at term, but confounders were not controlled for [14].

Semi-quantitative resistance indices such as PI, obtained from the UtA and UA, assess downstream vascular resistance, not placental volume blood flow. Doppler-assisted volume flow quantification in the UV, i.e. Q_UV,_ gives a more direct assessment of the actual fetoplacental blood flow [18], [19], [23]. Reduced normalized Q_UV_ is an early finding in fetal growth restriction [20], [38] and occurs in advance of significant changes in resistance measures in the UA [17], [22] or fetal biometry [20]. Our findings suggest that normalized fetoplacental volume flow is a more sensitive measure than UA resistance indices also when investigating effects of maternal distress on the fetoplacental circulation.

Using normalized, i.e. size-specific volume blood flow assessment in particular could help to understand the mechanisms behind the association between stress and reduced birthweight. Since fetoplacental volume flow is strongly associated with fetal size and growth, a reduced absolute flow in smaller fetuses is to be expected regardless of mechanism. However, if vasoconstrictive stress hormones cause a reduction in placental blood flow, as has been proposed [1], [3], [11], one would expect the fetus to receive less blood than appropriate for its size, i.e. a reduced normalized volume flow. If, on the other hand, reduced growth were caused by for example growth-inhibitory hormones such as glucocorticoids, one would expect to find fetoplacental volume blood flow proportionate to fetal size, i.e. an adequate normalized Q_UV_. Therefore, our results suggest that a reduction in fetoplacental volume flow may play a role in the possible association between certain types of maternal distress and fetal growth.

UA PI and Q_UV_ are known to correlate inversely, although not closely [23], [24]. In our study, a correlation with maternal distress was found only for Q_UVAC_, not for UA or UtA PI. This could be due to a combination of lack of power, low to moderate distress levels, and limited sensitivity of arterial resistance indices. However, if a slight increase in placental vascular resistance through vasoconstriction were on the pathway between stress and fetoplacental volume flow, controlling for UA PI in our regression models should have led to a reduction of statistical significance for the relation between intrusive distress and Q_UVAC_. On the contrary, after adjusting for UA PI, the significance of intrusion scores increased, and both variables contributed independently to predict Q_UVAC_. Therefore, we cannot identify increased vascular resistance as the probable pathway between distress and reduced fetoplacental blood flow.

Not all distress measures had an effect on Q_UVAC_. Partially, this could be due to the low scores for some of the distress measures. Specific types of maternal distress appear to have different effects on pregnancy outcomes [5], [7], [25]. Depressive symptom scores were very low in our study group, precluding the detection of an effect of this particular type of distress on blood flow measures. Pregnancy-specific stress or anxiety has been identified as a distinct type of anxiety [39] which has been found to be more strongly associated with unfavorable obstetric outcomes and infant development than anxiety or stress in general [3], [5], [40]. In our study, the IES referred specifically to thoughts and emotions about the health of the fetus. The intrusion subscale with its unbidden thoughts and emotions especially might describe a phenomenon similar to an important component of pregnancy-specific anxiety, i.e. worry or fear about whether the child is healthy. However, we are not aware of any study investigating such an overlap between IES and pregnancy-specific anxiety questionnaires.

The present study does not differentiate between current stress and chronic or repeated stress. This distinction would certainly have been of interest. The relatively low GHQ scores in our study suggest that most of the women had not been suffering from sustained severe distress. Previous studies have used the Spielberger State-Trait Anxiety Inventory (STAI), which assesses both State (i.e. current, situational) and Trait (i.e. dispositional) anxiety [11], [14]–[16]. While STAI is a general measure of anxiety, IES in our study focused specifically on distress related to the health of the fetus. Previous studies report that worries about “something being wrong with the baby” are highly prevalent throughout pregnancy [41], [42]. Therefore, we cannot rule out that the observed association between intrusive distress in third trimester and fetoplacental blood flow originated earlier in pregnancy, and could be due to sustained worry throughout pregnancy.

Our study was too small to detect the minor effects of maternal distress on birthweight reported previously [9], [10]. Therefore, it does not allow conclusions about the clinical significance of our findings. Our results need corroboration, but are consistent with the growing body of evidence suggesting that antenatal stress and anxiety, even at moderate levels, may have an effect on pregnancy and the offspring.

Our findings suggest a possible mechanism for a link between certain types of maternal distress and fetal growth, namely reduced fetoplacental volume blood flow. Since it is likely that any effect of maternal distress on fetal and child development is dose-dependent, it would be of interest to perform similar studies measuring fetoplacental volume blood flow in populations with high levels of depression or pregnancy worries, e.g. due to previous pregnancy complications.

## Supporting Information

Figure S1 **Schematic drawing of the placental circulation.** (Image Credit: Pernille Frese, Anne Helbig, Guttorm Haugen). Doppler ultrasound measurement sites. a) Uterine artery (UtA), carrying blood to the maternal compartment of the placenta. Assessment of vascular resistance in the uteroplacental circulation (pulsatility index (UtA PI) and diastolic notches). b) Umbilical artery, carrying blood from the fetus to the fetal compartment of the placenta. Assessment of vascular resistance in the fetoplacental circulation (UA PI). c) Intra-abdominal part of the umbilical vein (UV), with nutrient- and oxygen-rich blood flowing from the placenta to the fetus. Assessment of fetoplacental (i.e. umbilical vein) volume blood flow, normalized for fetal abdominal circumference (Q_UVAC_; ml/min/cm).
(TIF)

Figure S2 **Scatter plot showing correlation between IES intrusion scores and normalized umbilical vein blood flow (Q_UVAC_).** r(s), Spearman’s correlation coefficient; Q_UVAC_, umbilical vein blood flow normalized by fetal abdominal circumference (ml/min/cm).
(PDF)
