# Supplementary figures and images for: Does Antenatal Maternal Psychological Distress Affect Placental Circulation in the Third Trimester?
Source: PLoS One. 2013 Feb 20;8(2):e57071. doi: 10.1371/journal.pone.0057071 (PMC3577751; doi:10.1371/journal.pone.0057071)

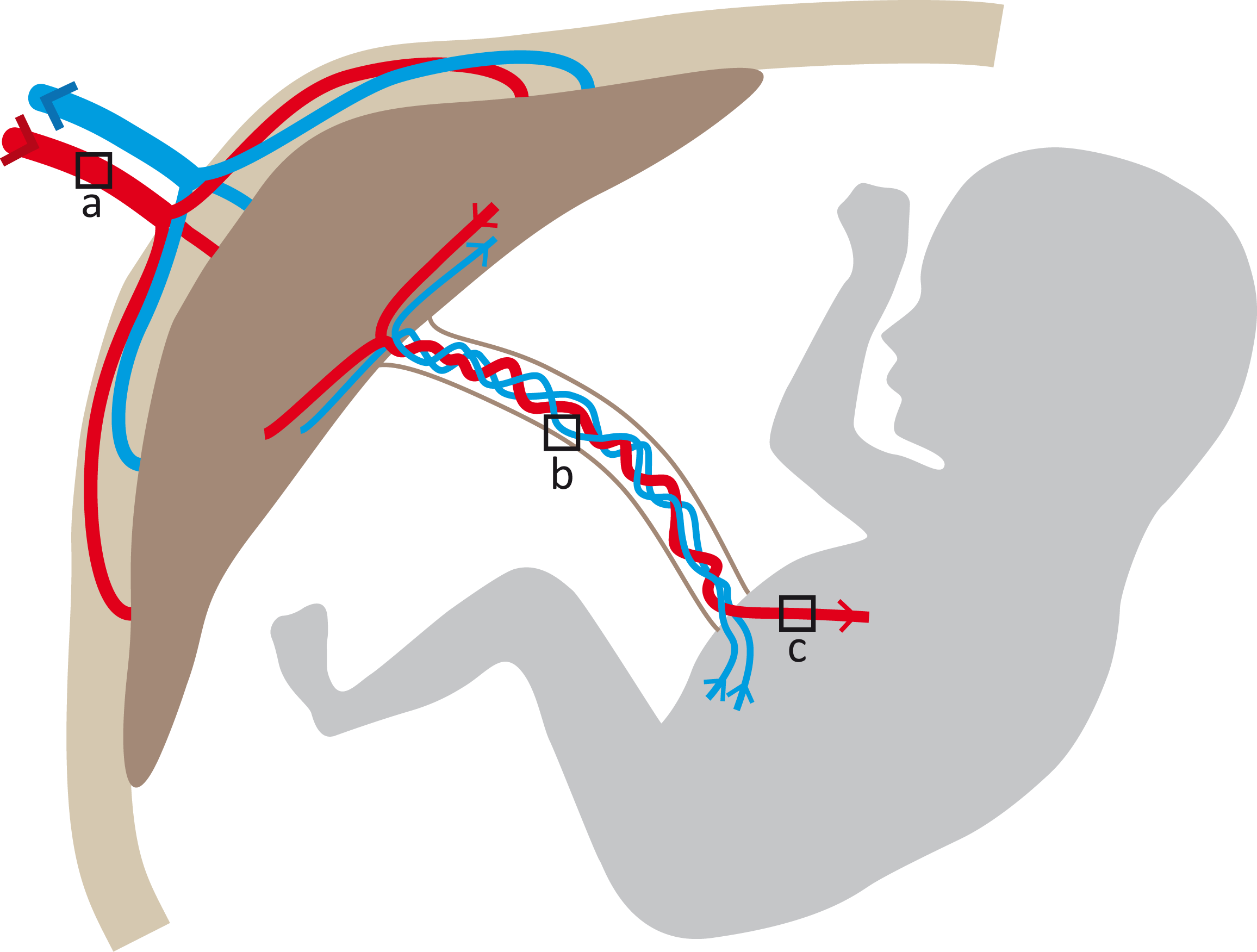

Supplement: Figure S1 — Schematic drawing of the placental circulation. (Image Credit: Pernille Frese, Anne Helbig, Guttorm Haugen). Doppler ultrasound measurement sites. a) Uterine artery (UtA), carrying blood to the maternal compartment of the placenta. Assessment of vascular resistance in the uteroplacental circulation (pulsatility index (UtA PI) and diastolic notches). b) Umbilical artery, carrying blood from the fetus to the fetal compartment of the placenta. Assessment of vascular resistance in the fetoplacental circulation (UA PI). c) Intra-abdominal part of the umbilical vein (UV), with nutrient- and oxygen-rich blood flowing from the placenta to the fetus. Assessment of fetoplacental (i.e. umbilical vein) volume blood flow, normalized for fetal abdominal circumference (QUVAC; ml/min/cm). (TIF) [file pone.0057071.s001.tif]

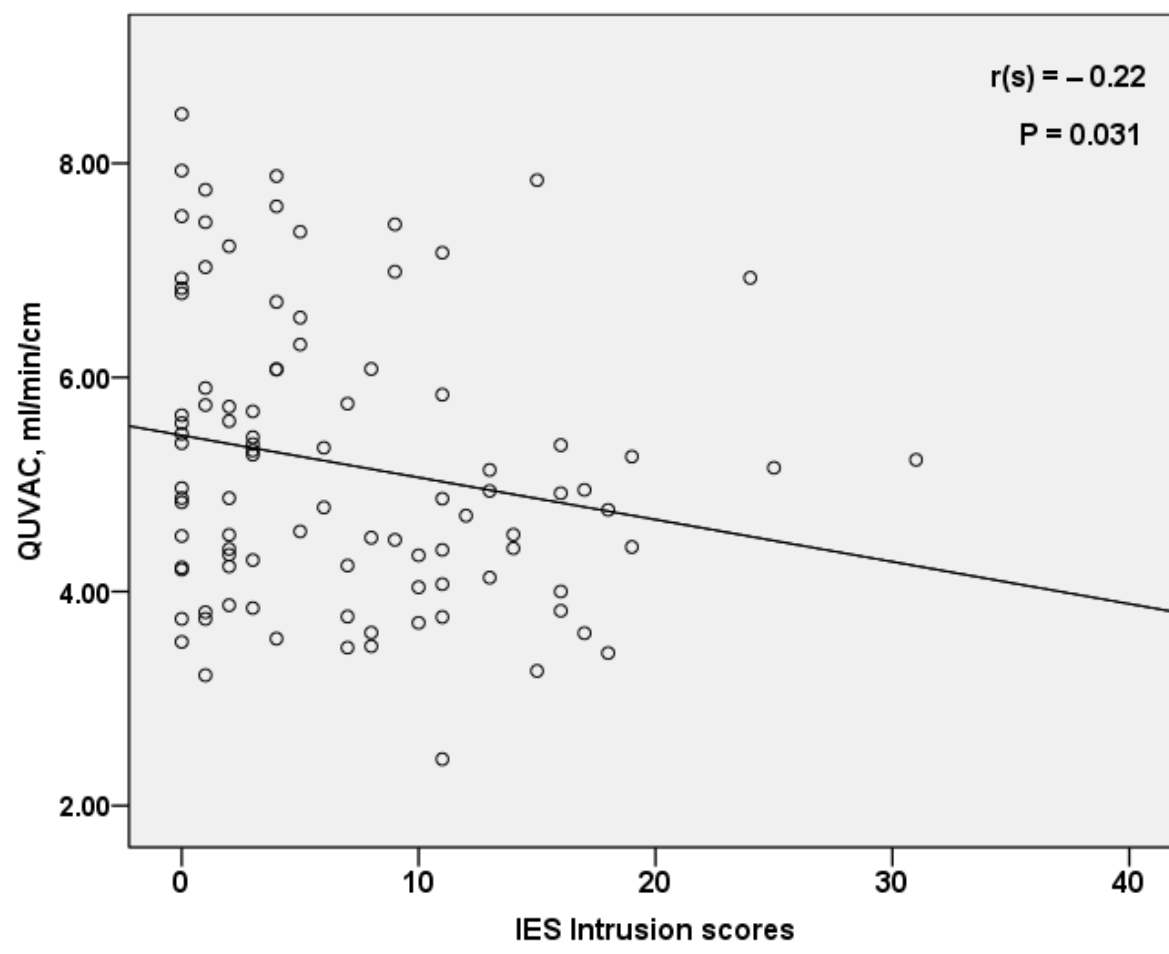

Supplement: Figure S2 — Scatter plot showing correlation between IES intrusion scores and normalized umbilical vein blood flow (QUVAC). r(s), Spearman’s correlation coefficient; QUVAC, umbilical vein blood flow normalized by fetal abdominal circumference (ml/min/cm). (PDF) [file pone.0057071.s002.pdf]
